# Fail-safe detection of threshold crossings of linear integrate-and-fire neuron models in time-driven simulations

**DOI:** 10.1186/1471-2202-12-S1-P229

**Published:** 2011-07-18

**Authors:** Susanne Kunkel, Moritz Helias, Markus Diesmann, Abigail Morrison

**Affiliations:** 1 Functional Neural Circuits Group, Faculty of Biology, Albert-Ludwig University of Freiburg, Germany; 2 Bernstein Center Freiburg, Albert-Ludwig University of Freiburg, Germany; 3 RIKEN Brain Science Institute, Wako, Japan; 4 Institute of Neuroscience and Medicine (INM-6), Computational and Systems Neuroscience, Research Center Jülich, Germany; 5 RIKEN Computational Science Research Program, Wako, Japan

## 

The characteristics of time-driven simulation are a fixed-size simulation step and a fixed-size communication interval [1]. The former defines update-and-check points, which are the discrete points in time when all neurons update their state variables and check for a super-threshold membrane potential. The latter defines the discrete points in time when all neurons communicate their spikes. The communication interval is a multiple of the simulation step size and limited only by the minimum synaptic transmission delay in the network.

The time-driven environment of the simulator NEST [2] provides an 'on-grid' and an 'off-grid' framework that handle spikes differently. In the on-grid framework, spikes are incorporated, detected and emitted only at the pre-defined update-and-check points. In the off-grid framework, spikes can be incorporated and emitted at any point in time [3]. For each neuron the arrival times of incoming spikes introduce additional update-and-check points. Hence, the simulation step can be increased up to the size of the communication interval. The detection of a threshold crossing can only take place at a check point, but the timing of the referring spike is estimated with precision limited only by the limits of double representation.

In general, a time-driven simulator that supports the off-grid framework performs neural network simulations with the same precision and faster than an event-driven simulator [4]. However, time-driven simulation still bears the risk of missing a threshold crossing as a very brief excursion of the membrane potential above threshold may not be detected at the next check point. In the off-grid framework, this problem is more pronounced in networks with low connectivity and strong coupling as well as in the case of low firing rates. The on-grid framework is even more affected due to fewer check points and the synchronized arrival of spikes.

Here, we present algorithms which are guaranteed to detect all threshold crossings by supplementing the standard test for a super-threshold membrane potential at each check point and that exploit the information about the neuronal state at nearby check points. These additional tests need to be invoked whenever the membrane potential is sub-threshold, which means at virtually all check points. We develop sub-tests of increasing complexity and specificity, starting with simple sifting methods and ending up with a complex expression that faithfully indicates the existence of a threshold crossing between the last and the current check point. An analysis of the test specificities and computational costs results in a cascade of tests which locates all threshold crossings at a low computational cost.
